# Ellagic Acid Induces in vitro Alkalinisation of the Digestive Vacuole in Drug-Sensitive Plasmodium falciparum Strain

**DOI:** 10.21315/mjms2022.29.4.5

**Published:** 2022-08-29

**Authors:** Nur Hazirah Muchtar, Nik Nor Imam Nik Mat Zin, Fatin Sofia Mohamad, Nurhidanatasha Abu-Bakar

**Affiliations:** School of Health Sciences, Universiti Sains Malaysia, Kelantan, Malaysia

**Keywords:** ellagic acid, phenolic compound, Plasmodium falciparum, pH, digestive vacuole, proton pump

## Abstract

**Background:**

Malaria is one of the leading causes of death worldwide caused by parasites of the genus *Plasmodium*. The reduced efficacy of the mainstay antimalarial drugs due to the widespread of drug-resistant *Plasmodium falciparum* (*P. falciparum*) necessitates an effort to develop novel antimalarial drugs with new targets. The effects of a phenolic compound, ellagic acid, against the malaria parasite have previously been reported. This present study aimed to evaluate the effect of ellagic acid on pH of the *P. falciparum* digestive vacuole.

**Methods:**

The antimalarial potential of ellagic acid against the chloroquine-sensitive strain (3D7) of *P. falciparum* was assessed by using a malarial SYBR Green 1 fluorescence-based (MSF) assay. The effect of different concentrations of ellagic acid on the pH of the parasite’s digestive vacuole at mid-trophozoite stage was examined by using a ratiometric pH indicator, fluorescein isothiocyanate (FITC)-dextran on the flow cytometry.

**Results:**

The result of the MSF assay showed that ellagic acid has an antimalarial activity (half-maximal inhibitory concentration [IC_50_] = 1.85 ± 4.57 nM) at par with a standard drug, artemisinin (IC_50_ = 1.91 ± 5.41 nM). The pH of the digestive vacuole of ellagic acid-treated parasites was significantly changed (pH values ranged from 6.11 to 6.74) in a concentration-dependent manner as compared to untreated parasites (*P* < 0.001). A similar effect was shown by the parasites treated with a standard proton pump inhibitor, concanamycin A.

**Conclusion:**

These findings suggest that ellagic acid might have altered the digestive vacuole pH through the inhibition of proton pumps that regulate the acidification of this organelle. Overall, this study provides a valuable insight into the potential of ellagic acid as a promising antimalarial candidate with a novel mechanism of action.

## Introduction

Malaria is a mosquito-borne disease that imposes significant health and socioeconomic impacts on humans. This disease contributes to the high rate of global infectious disease-related morbidity and mortality with 409,000 deaths from 229 million cases reported in 2019 (1). Out of the five *Plasmodium* species causing human malaria, *Plasmodium falciparum* (*P. falciparum*) is the most virulent and responsible for the highest disease burden, accounting for over 90% of the malaria deaths worldwide (1). Although a decline in the incidence of malaria results from the removal of mosquito breeding sites with insecticides, the use of long-lasting insecticide-treated nets and indoor residual spraying (2), an effective administration of antimalarial drugs can significantly reduce the overall malaria-related morbidity and mortality (3). The emergence of resistance to artemisinin-based combination therapies (ACTs) as the current frontline treatments has, however, threatened the malaria control efforts (4). Given this drawback and the absence of protective malaria vaccines, the discovery of new antimalarial agents particularly with novel mechanisms of action is urgently needed.

Medicinal plants constantly become the source of antimalarial drug candidates. They are rich in phytochemicals such as alkaloids, terpenes, flavanones and phenolics that are highly efficacious in the treatment of malaria (5–7). Active metabolites, quinine and artemisinin, which are one of the most successful antimalarial drugs, are derived from these classes of compounds (8). Ellagic acid (3,3′,4,4′-tetrahydroxydiphenic acid dilactone) is a naturally occurring phenolic compound found in certain oak species (9), pomegranate (10), longan (11) and lychee (12). This compound has been reported to have a number of biological activities such as anticarcinogenic (13), anti-inflammatory (14) and antimalarial properties (15–17). The abundance of pyrogallol (18–20) and ellagic acid (21–23) in *Quercus infectoria*, an oak species, might contribute to the activity against the malaria parasite (24–25). Ellagic acid has been postulated to have the antimalarial effect through impairment of the haemoglobin degradation and β-haematin formation in the parasite’s digestive vacuole (15–16, 26).

The digestive vacuole is the site of action of several existing antimalarial drugs (27–29). Physiologically, the digestive vacuole of *P. falciparum* comprises proteases such as plasmepsins and falcipains that function optimally at low pH values, ranging from 4.0 to 5.5 (30–32), which are similar to the pH values 3.7–6.5 of the digestive vacuole (33–34). The digestive vacuole needs to maintain its acidic pH condition to facilitate efficient haemoglobin degradation and heme detoxification (35). Furthermore, the acidification of the digestive vacuole is tightly regulated by proton pumps, the vacuolar H^+^-ATPase (V-type H^+^-ATPase) and pyrophosphatase (V-type H^+^-pyrophosphatase) (36–38). Hence, the in vitro antimalarial effect of ellagic acid against *P. falciparum* through the alteration of the digestive vacuole pH was investigated in this study.

## Methods

### The Malaria Parasite in vitro Culture

*P. falciparum* (3D7 strain) was maintained in RPMI 1640 medium (Gibco, USA) and type O^+^ human erythrocytes at 2% haematocrit by using an established protocol (39). Ring stage parasites (5% parasitemia) were synchronised by using 5% D-sorbitol (Sigma-Aldrich, USA) (29) as employed in the subsequent assay after 2 h post-synchronisation.

### The Malarial SYBR Green 1 Fluorescence-Based Assay

The antimalarial activity of ellagic acid was determined by using the malarial SYBR Green 1 fluorescence-based (MSF) assay (40). The stock solution of ellagic acid (11 mM) (Sigma-Aldrich, USA) was diluted in RPMI 1640 medium at 10 concentrations of two-fold serial dilutions into 96-well microtiter plates. Artemisinin (Sigma-Aldrich, USA) was used as a standard drug, whereas infected erythrocytes without drug treatment served as a negative control. Aliquots (20 μL) of different concentrations of the compounds were transferred into other plates and added with suspensions (180 μL) of synchronised ring stage parasites (2% parasitemia, 2% haematocrit). After incubation for 48 h at 37 °C in a 5% CO_2_, the cell suspensions in the plates were stained with 20× SYBR Green 1 solution (20 μL) (Sigma-Aldrich, USA) for 1 h at room temperature (41). The total fluorescence signal was measured with a microplate reader at the excitation and emission wavelengths of 490 nm and 530 nm, respectively and used to calculate the parasite inhibition (%) of each concentration. Half-maximal inhibitory concentration (IC_50_) values of the drugs were determined by probit regression analysis with GraphPad Prism software version 8.

### The FITC-Dextran Encapsulation into Erythrocytes

Packed erythrocytes were lysed in 2.25 volumes of ice-cold haemolysis buffer (5 mM sodium phosphate, pH 7.5) supplemented with Mg-ATP (1 mM) in the presence of fluorescein isothiocyanate (FITC)-dextran (25 μM) (Sigma-Aldrich, USA; 10 kDa) (27). After incubation for 10 min to allow the encapsulation of the probe into the cells, the isotonic condition of the erythrocytes was restored by using resealing buffer A before incubation with resealing buffer B for 20 min at 37 °C. Cell suspensions were washed twice with resealing buffer B and once with RPMI 1640 medium. Erythrocytes resealed with FITC-dextran in RPMI 1640 medium were used in the subsequent assay.

### The Digestive Vacuole pH Measurement

The pH calibration curve of FITC-dextran was generated to measure pH of the digestive vacuole (28, 42). Resealed erythrocytes containing FITC-dextran (2% haematocrit) were suspended in different buffers (20 mM) (MES, pH 4.0, 5.5 and 6.0; NaH_2_PO_4_, pH 6.5, 7.0, 7.5 and 8.0; TRIS, pH 9.0) supplemented with 150 mM NaCl and in the presence of 10 μM ionophore, carbonyl cyanide m-chlorophenylhydrazone (CCCP; Sigma-Aldrich, USA). FITC-dextran was excited by using a 488 nm argon ion laser of FACSCanto^TM^ II flow cytometer (Becton Dickinson) and its fluorescence intensity was collected at FITC/green (530 nm) and phycoerythrin (PE)/yellow (585 nm) channels. The data were analysed using FCS Express 5 flow cytometry software (De Novo Software). The cell population was gated based on their side scatter (SSC) and forward scatter (FSC) profiles and an additional gate was established based on the fluorescence intensity of FITC-dextran at green and yellow. The peak of the fluorescence intensity (R_gy_) for both green and yellow channels was obtained from the histograms from which the ratio of green/yellow R_gy_ was measured and plotted as a function of the pH.

Synchronised mid-trophozoite stage parasites (~34-h post-invasion) grown in resealed erythrocytes containing FITC-dextran (5% parasitemia, 2% haematocrit) were treated with ellagic acid at different concentrations based on the IC_50_ value obtained: 0.93 nM (0.5 × IC_50_), 1.85 nM (1.0 × IC_50_) and 3.70 nM (2.0 × IC_50_). A standard proton pump inhibitor, concanamycin A (75 nM) (Sigma-Aldrich, USA) was used as a positive control and untreated parasites as a negative control. After 4-h incubation, infected erythrocytes were selectively permeabilised with 0.035% saponin (Sigma-Aldrich, USA) to permeabilise the erythrocyte plasma membrane (EPM) and the parasitophorous vacuolar membrane (PVM), releasing FITC-dextran in the erythrocyte cytoplasm and allowing only FITC-dextran entrapped in the digestive vacuole for the digestive vacuole pH measurement (28, 36). Saponin-permeabilised parasites were washed twice in extracellular saline medium before measuring the FITC-dextran fluorescence intensity by using flow cytometry. Changes in pH of the digestive vacuole were measured by interpolating R_gy_ in the pH calibration curve of FITC-dextran.

### Statistical Analysis

All experiments were conducted in triplicates (*n* = 3) on three independent occasions and analysed with GraphPad Prism software version 8. Mean values were expressed as mean (standard deviation [SD]). The data were tested for normality before proceeding to one-way analysis of variance (ANOVA), followed by Dunnett multiple comparisons at 95% confidence intervals (comparison between treated groups and control groups) using the IBM SPSS Statistics version 20. A *P*-value less than 0.05 was considered statistically significant.

## Results

**Figure 1:**
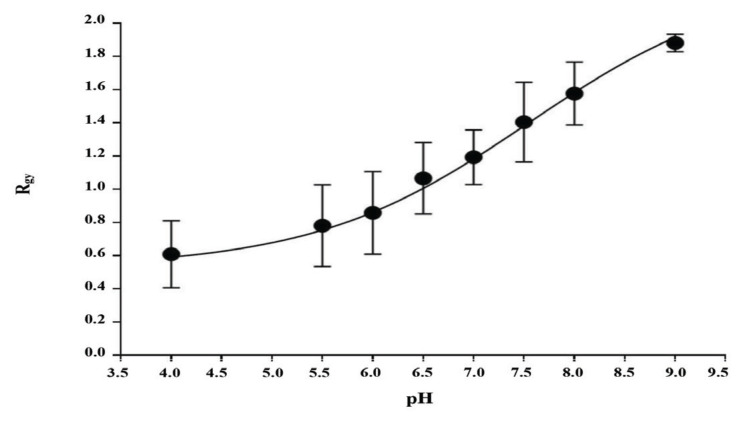
A standard pH calibration curve of FITC-dextran. A FITC-dextran pH calibration curve was constructed by suspending resealed erythrocytes in buffers of different pH (4.0–9.0) in the presence of an ionophore, carbonyl cyanide m-chlorophenylhydrazone (CCCP). The fluorescence intensity was collected at green and yellow channels by flow cytometry. The ratio of green/yellow fluorescence intensity (R_gy_) was plotted on the *y*-axis against the pH on the *x*-axis. The dose-response curve was fitted by non-linear regression with GraphPad Prism (*R*^2^ = 0.9982). The data are expressed as mean (SD) derived from three independent experiments done in triplicate

The MSF assay shows that ellagic acid exhibited an antimalarial activity (IC_50_ = 1.85 ± 4.57 nM) against the 3D7 parasite at par with artemisinin (IC_50_ = 1.91 ± 5.41 nM). The mid-trophozoite stage parasite-infected erythrocytes were then treated with three different concentrations of ellagic acid (0.93 nM, 1.85 nM and 3.70 nM) selected based on the IC_50_ value previously obtained. Furthermore, the pH change of the digestive vacuole of the parasites treated with ellagic acid for 4 h was observed and compared to the untreated parasites. As shown in Figure 1, the R_gy_ in the pH calibration curve of FITC-dextran provides a measure of digestive vacuole’s pH. The R_gy_ value increased (ranged from ~0.6–1.9) with increasing pH (4.0–9.0) with pK_a_ of ~5.8 (the inflection point in the curve), indicating that FITC-dextran is sensitive to pH.

**Figure 2:**
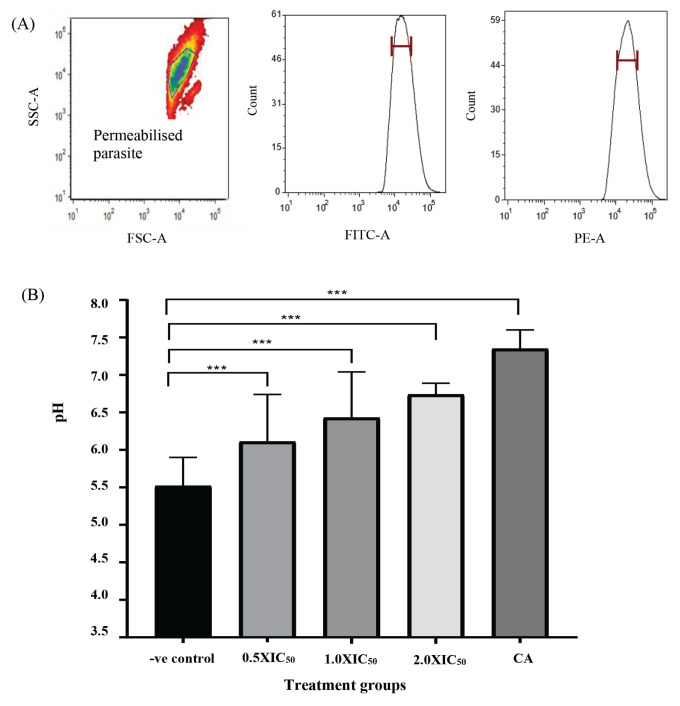
Analysis of the pH of the digestive vacuole of *P. falciparum* after treatment with different concentrations of ellagic acid. (A) Representative scatter and fluorescence intensity profiles of the saponin-permeabilised parasite population at FITC/green and PE/yellow channels. (B) The effect of ellagic acid on pH of the digestive vacuole was investigated by using three different concentrations: 0.5 × IC_50_ (0.93 nM), 1.0 × IC_50_ (1.85 nM) and 2.0 × IC_50_ (3.7 nM). The untreated mid trophozoite stage parasite was used as a negative control, while concanamycin A (CA; final concentration of 75 nM) was used as a positive control. The ratio (R_gy_) was calculated for each treatment and converted to a pH value by means of the generated standard calibration curve in Figure 1. Note: ***P < 0.001 was considered as statistically significant

Prior to digestive vacuole pH measurement, the parasites were isolated with saponin to permeabilise the EPM and PVM, allowing only the digestive vacuole-located fluorescence intensity to be measured by the flow cytometer. Based on the established gating of saponin-permeabilised parasites (Figure 2A, left panel), the digestive vacuole pH values treated with 0.93 nM, 1.85 nM and 3.70 nM of ellagic acid resulted in 0.59, 0.91 and 1.22 pH unit higher, respectively than that of the untreated parasites (pH = 5.52 ± 0.38) (*P* < 0.001) (Figure 2B). The transition of the digestive vacuole pH from acidic to alkaline (pH = 7.35 ± 0.25) was also observed in the population of viable parasites treated with a standard proton inhibitor, concanamycin A.

## Discussion

Ellagic acid has been shown to possess a number of biological and pharmacological activities (43–46). The compound exhibited a promising antimalarial activity in vitro, which is in agreement with a report by Garcia-Alvarez et al. (17) that ellagic acid was active against a wide range of *P. falciparum* strains with IC_50_ values in the nanomolar range. Notably, ellagic acid also has an in vivo antimalarial activity against *P. vinckei petteri* (17), *P. berghei* (47) and *P. yoelli* (15). The compound was found to be more active at mature stage parasites when most of the hemoglobin-rich host cell cytoplasm was ingested and digested (15). Artemisinin, the standard antimalarial drug showed an IC_50_ of 1.91 ± 5.41 nM that is consistent our previous studies (48–50), confirming the validity of the assay.

Only a few studies of the effect of ellagic acid on the malaria parasite’s digestive vacuole are evident in the literature. Thus, the effect of ellagic acid with an emphasis on pH on the digestive vacuole was investigated to determine its possible mechanism of action. The present study reported an increase of the digestive vacuole pH following treatment with ellagic acid. Meanwhile, the digestive vacuole pH of untreated parasites was acidic, which is consistent with other studies using the same probe (27–28, 34). The treatment of the parasites with a V-type H^+^-ATPase inhibitor, concanamycin A, resulted in the alkalinisation of the digestive vacuole. This suggests that the digestive vacuole proton pumps might be active in situ. This is in agreement with previous studies showing the capability of concanamycin A to disrupt pH regulation by preventing the transportation of H^+^ into the digestive vacuole and out of the parasite across the parasite plasma membrane (51–52). Therefore, the inhibition of the proton pumps that causes the pH alteration of the digestive vacuole might become a possible mechanism of ellagic acid action.

Ellagic acid activity has also been linked to the inhibition of plasmepsin II responsible for the digestion of haemoglobin and the impairment of β-haematin formation in the digestive vacuole (15–16, 26). The process of β-haematin formation could be inhibited by acting on heme monomers to coordinate their oxidation. Some phenolic compounds have exhibited the inhibitory effect on the heme polymerisation and β-haematin/hemozoin formation occurred particularly within the digestive vacuole. There is a possibility that ellagic acid might also have an effect on proton pumps located on the digestive vacuole’s membrane, which results in the alteration of the acidic pH of this organelle. Hydroxychloroquine, a standard antimalarial drug has been shown to accumulate in the digestive vacuole along a pH gradient and inhibit the degradation of cargo derived from endocytosis or autophagy pathway. This process is executed by increasing the digestive vacuole pH, thus preventing the activity of the digestive vacuole enzymes (53).

The acidification of the parasite digestive vacuole is vital for the occurrence of various biological processes. It has been suggested that changes in the acidic environment might interfere with haemoglobin degradation and subsequently heme detoxification (48). This will inhibit the growth and proliferation of the parasite. Several studies described that the subtle pH changes in other acidic organelles, such as the lysosome caused the significant decrease in lysosomal enzyme activities, thus causing various pathological alterations (54–56). The increase of only a few tenths of a pH unit might have a major impact on the lysosomal function, whereas the organelle needs to maintain its acidic pH condition as in the digestive vacuole for the optimal activity of proteases (57). As the V-type H^+^-ATPase is localised within a membrane-enclosed organelle, ellagic acid might probably disrupt the pumping function and therefore alter the digestive vacuole pH, which was evidenced in this study. The important role that the V-type H^+^-ATPase plays in regulating the physiological pH of the digestive vacuole enables a primary explanation for the antimalarial potential of ellagic acid. Therefore, the postulated mechanism of action of ellagic acid, which caused the pH alteration in the digestive vacuole is via the inhibition of the V-type H^+^-ATPase.

## Conclusion

In summary, this in vitro study unveiled the promising antimalarial activity of ellagic acid against *P. falciparum* and the compound was shown at par with artemisinin. It is suggested that ellagic acid increased the digestive vacuole pH of the treated parasites probably by serving as an inhibitor of the proton pumps localised on the membrane of the digestive vacuole. A similar digestive vacuole pH alteration was shown by concanamycin A. This can be confirmed by performing isobologram analysis involving the interaction between ellagic acid and concanamycin A, the specific V-type H^+^-ATPase inhibitor or NaF, the specific V-type H^+^-pyrophosphatase inhibitor to confirm the target of ellagic acid.
